# Low-Dose Intratympanic Gentamicin for Unilateral Ménière‘s Disease: Accuracy of Early Vestibulo-Ocular Reflex Gain Reduction in Predicting Long-Term Clinical Outcome

**DOI:** 10.3389/fneur.2022.808570

**Published:** 2022-03-18

**Authors:** Ricardo Wegmann-Vicuña, Raquel Manrique-Huarte, Diego Calavia-Gil, Eduardo Martín-Sanz, Pedro Marques, Nicolas Perez-Fernandez

**Affiliations:** ^1^Department of Otorhinolaryngology, Clínica Universidad de Navarra, Pamplona, Spain; ^2^Department of Otorhinolaryngology, Hospital Quirónsalud, Barcelona, Spain; ^3^Department of Otolaryngology, University Hospital of Getafe, Madrid, Spain; ^4^Department of Medicine, School of Biomedical Sciences and Health, Universidad Europea de Madrid, Madrid, Spain; ^5^Department of Otorhinolaryngology, S. João Hospital Centre, Porto, Portugal; ^6^Department of Otorhinolaryngology, Clínica Universidad de Navarra, Madrid, Spain

**Keywords:** vertigo, Ménière's disease, inner ear, ototoxicity, hearing loss

## Abstract

**Background:**

The number of intratympanic gentamicin (ITG) injections needed to achieve vertigo control in patients with intractable Ménière's disease (MD) may vary from a single dose to several instillations. Changes in different vestibular test results have been used to define an endpoint of treatment, including the decrease of the vestibulo-ocular reflex (VOR) gain elicited by the head-impulse test.

**Objective:**

To assess the accuracy of the VOR gain reduction after horizontal canal stimulation, as measured with the video head-impulse test (vHIT) 1 month after the first intratympanic injection, in predicting the need for one or more instillations to control vertigo spells in the long term.

**Methods:**

The VOR gain reduction was calculated in 47 patients submitted to (ITG) therapy 1 month after the first instillation.

**Results:**

Single intratympanic treatment with gentamicin has a 59.6% efficacy in vertigo control in the long term. Hearing change in the immediate period after treatment (1 month) is not significant to pre-treatment result and is similar for patients who needed multiple doses due to recurrence. Chronic disequilibrium and the need for vestibular rehabilitation were less frequent in patients with a good control of vertigo with just one single injection of gentamicin. A fair accuracy was obtained for the VOR gain reduction of the horizontal canal (area under the curve = 0.729 in the Receiver Operating Characteristic analysis) in predicting the need for one or more ITG.

**Conclusions:**

Single intratympanic treatment with gentamicin is an effective treatment for patients with MD. That modality of treatment has very limited damaging effect in hearing. The degree of vestibular deficit induced by the treatment is significant as measured by the reduction in the gain of the VOR but not useful for prognostic purposes.

## Introduction

Ménière's disease (MD) is an idiopathic inner ear disorder characterized by episodic vertigo spells lasting from 20 min to 12 h, fluctuating low-frequency hearing loss during the initial spells with associated aural fullness, and tinnitus (1). Current therapies aim to control vestibular symptoms with conservative means, namely, low-sodium diet, diuretics, and vestibular suppressants. For those patients who do not respond, alternatives for alleviating the symptoms include surgical interventions like labyrinthectomy, vestibular neurectomy, or less invasive therapies like intratympanic injection of gentamicin or steroids.

The use of gentamicin delivered intratympanically (ITG) is an effective treatment (2) because it produces a variable degree of vestibular function ablation (3), and this reduces the sensitivity of the vestibular periphery sensors and clinical symptoms. Unfortunately, the amount of damage to the inner ear cannot be predicted, and a trend toward less ototoxic damage has been the interest in current protocols of treatment. As such, different number and frequency regimens and different drug concentrations have been proposed to achieve better vertigo control with the lowest risk of hearing loss and residual instability (4, 5). This can be achieved by using lower doses of gentamicin and fewer injections with longer time intervals between injections. Using a single-shot therapy with an *on-demand* protocol, complete or substantial control of vertigo attacks ranges between 80 and 90%, and complete vertigo control can be accomplished with a single injection of gentamicin in 45–54% of the patients (6–8). It is well tolerated by patients and the amount of post-treatment instability is reduced.

In this modality of treatment, gentamicin gets into the inner ear mainly through the round window membrane and the oval window. It has been shown in the experimental situation that the distribution of gentamicin either in the scala tympani or in the scala vestibuli will be the result of different interactions (9). In experimental studies using gentamicin locally applied in the middle ear it has been shown that it attacks differentially type I receptors in the vestibular end-organs and the dendrites of primary afferent neurons (10). As a result, the vestibulo-ocular reflex (VOR) gain measured, with a scleral search coil or with the video head-impulse test, will be reduced as the response in the caloric test. This gain reduction has been associated with a decrease in the rate of vertigo attacks in patients with MD (11).

Several methods have been used to define a safety endpoint in this modality of treatment, including modification of bedside testing (spontaneous nystagmus, post-head-shaking nystagmus, and refixation saccades on the head impulse test) or vestibular laboratory test results [decreased VOR gain on the quantitative head thrust test, abolished caloric, and vestibular evoked myogenic potential (VEMP) responses]. However, there is yet no consensus on the optimal dose and concentration of gentamicin to be delivered to obtain a significant clinical result. In a previous report, Marques et al. proposed an endpoint of treatment, such that a horizontal canal VOR reduction of at least 17.8% [area under the curve (AUC) of Receiver Operating Characteristic (ROC) curve = 0.843] and a posttreatment asymmetry of at least 7% in the horizontal semicircular canal (AUC = 0.861) was associated with a good vertigo control, which meant that there was no need of further injections during a mean period of 21 months of follow-up (12). However, 13.6% of the patients initially treated with one single ITG injection in that study presented recurrence of vertigo in the long-term follow-up, and the effectiveness of a single dose of gentamicin for vertigo control fell from 71 to 58% (unpublished data).

The first follow-up after ITG is 1 month in different studies based on the short-term dynamics of the vestibulo-toxic effect of ITG. Previous researchers have shown that throughout the first month, there is a significant reduction in the gain of the VOR which stabilizes 1 month after treatment (13).

Our study aimed to assess the following aspects pertinent to the result of the treatment: (1) The effectiveness of one-single dose of gentamicin for long-term vertigo control, (2) How accurately immediate VOR gain reduction (as measured 1 month after the first injection) differentiates patients that need a further injection to achieve long-term vertigo control, i.e., does that change in VOR predict clinical course during the follow-up?, (3) Are there significant short-term changes in hearing after the first ITG treatment?, and (4) Is vestibular rehabilitation more frequently indicated after one or multiple doses of ITG?.

## Materials and Methods

### Subjects

We retrospectively analyzed 47 patients admitted to our clinic who were diagnosed with unilateral definite MD according to diagnostic criteria (1) from August 2011 to July 2017; some of these patients were included in a previous study (12). All patients underwent a complete neurotologic examination and were treated with ITG because of recurrent vertigo attacks which did not respond to conventional medical treatment (salt restriction, diuretics, and/or betahistine). Inclusion criteria were unilateral definite MD, video head-impulse test done before (usually the same day or no longer than a week before treatment) and 1 month after ITG treatment, no evidence of MD and serviceable hearing in the non-affected ear, and no symptoms or signs suggesting central nervous system involvement. All patients were re-evaluated at 1, 3, 12, and 24 months and then annually after the first gentamicin injection. During data acquisition, the following parameters were collected retrospectively: age and sex of the patient, disease duration, number of vertigo spells in the 6 months before treatment, time from the last attack, time of follow up, Tumarkin's attacks, audiometric findings, and migraine history. Additionally, we assessed 6 patients (here defined as untreated) diagnosed with definite unilateral MD without current medication, at baseline and 1 month after. We compared the VOR gain values and hearing test results with those of the patients treated intratympanically to find out to what extent the results of treated and untreated subjects are different and not time-dependent or fluctuant. These patients were selected upon appearance at the hospital and not considered to be age/sex-matched.

### Methods

#### Clinical Evaluation and Bedside Testing

All patients underwent a complete clinical assessment and bedside testing which consisted of microscopic examination of the eardrum, routine otolaryngological examination, search for spontaneous nystagmus and head-shaking nystagmus under Frenzel's glasses, clinical head impulse test, and positional nystagmus. The Dizziness Handicap Inventory (DHI) was used to evaluate the self-perceived handicapping effects of dizziness; the version used was taken from the original (14) and validated to the Spanish language (15).

#### Auditory and Vestibular Evaluation

Audiometric findings were reported in terms of the mean pure-tone average (PTA), calculated on air conduction thresholds at 0.5, 1, 2, and 3 kHz of the symptomatic or affected ear and the asymptomatic ear or non-affected ear. Patients were classified into four stages according to the American Academy of Otolaryngology–Head and Neck Surgery (AAO-HNS) (1).

Vestibular testing was performed with the vHIT (ICS Impulse, Otosuite V 4.0, GN Otometrics, Taastrup, Denmark) for the six semicircular canals, with 20 impulses in each direction according to the canal plane. The parameter evaluated was the VOR mean gain and the presence of refixation saccades, namely, overt and covert saccades. Gain is obtained from the gain value after each of the impulses is performed and is automatically provided for all paired semicircular canals assessed. Data were evaluated from the symptomatic (Gs) and asymptomatic (Gas) ears. Due to modifications in the hardware, horizontal canals were examined in 53 patients, and data from the vertical canals were obtained from 43 patients (38 in the treatment group and 5 in the untreated group).

#### Treatment

The medication used was gentamicin sulfate (40 mg/ml) buffered with sodium bicarbonate to pH 6.4 and a final concentration of 26.7 mg/ml, and 0.5–1.5 mL (1.06 ± 0.31) of the gentamicin solution was injected slowly in the affected ear through the posterior-inferior quadrant of the tympanic membrane. Care was taken to observe the progressive filling of the middle ear and of the round window niche when possible. The patients were asked to lie in a supine position with the affected ear for up to 30 min and were encouraged not to swallow.

The study was performed according to the following protocol, as previously approved by the Ethics and Medical Deontology Commission at our hospital. All patients that fulfilled the inclusion criteria and signed the corresponding informed consent received the first injection of gentamicin. Patients were then instructed to inform us of any symptoms that developed during the first month after the ITG injection. When necessary, vestibular suppressants were prescribed to treat vestibular symptoms. At the time of the first follow-up visit 1 month after the ITG injection, the clinical status of the patient was reviewed, and bedside vestibular examination, vHIT, and audiometric assessment were performed. After the first follow-up visit, all the patients were instructed to record any vertigo spell resembling those experienced before the treatment in case of chronic disequilibrium impacting daily activities. When new attacks developed, a decision was then made to treat the patient with another injection of gentamicin or with vestibular suppressants. In case of no more vertigo crises but chronic disequilibrium with a high-level handicap as defined by a DHI score >54, then the patient was treated with vestibular rehabilitation. Telephonic contact was maintained with every patient (in between follow-ups at the hospital) to assess any vertigo recurrence. For patients without vertigo recurrences, follow up at the end of the study (2 years) was made mostly by telephonic and/or e-mail contact.

### Statistical Analysis

For demographic analysis, differences between normally distributed data were assessed with Student's *t-*test or the one-way ANOVA test. For non-normally distributed data, the Wilcoxon test or the Kruskal-Wallis test was performed. Median and interquartile range (25th and 75th percentiles) are shown for not normally distributed data. Otherwise, mean and standard deviation are shown. Differences between percentages were determined by using Fisher's exact test.

According to the number of injections needed to achieve vertigo control, patients were classified into two treatment groups: one group treated with one single dose of ITG (group 1) and another group who needed during follow-up a subsequent or more ITG injections (group 2). The untreated group was designated as group 0.

The results of the vestibular evaluation were analyzed in terms of VOR gain reduction according to the formula: [(pre-treatment gain – post-treatment gain)/(pre-treatment gain)]^*^100.

Both data were combined such that patients were classified as “unexpected” when, in group 1, gain reduction was lower than 17.8% or, in group 2, >17.8%, Data were classified as “expected” when, in group 1, gain reduction was >17.8% or, in group 2, lower than 17.8%. A chi-square test was performed to compare the number of expected and unexpected results between treatment groups.

A description of the time course of recurrent vertigo was performed with the Kaplan-Meier survival analysis which allows quantification of the percentage of patients who had adequate control of their vertigo using the need for a second injection as a definition of “failure.” ROC curve analysis was performed for vestibular function (VOR gain) reduction regarding treatment outcome. A two-way factorial mixed ANOVA design was performed for VOR gain reduction as dependent variable, treatment groups as between-subjects factors, and ear and time as within-subjects factors. Differences between factors have been tested using contrasts. Cohen's *D* was calculated for assessing the size effect if considered clinically relevant.

All analyses were performed using the STATA 15.0 statistical software (StataCorp, College Station, TX, USA). A value of *p* < 0.05 was considered statistically significant. For interactions terms, a value of *p* < 0.1 has been considered statistically significant.

## Results

### Patients

The study included 47 subjects (28 women and 19 men) with definite MD that belong to the group of patients treated with ITG and 6 patients in the untreated group. There were 11 additional patients (4 in group 1 and 7 in group 2) not included because the first follow-up was not in the expected period of time (1 month) after treatment. There were 6 patients (2 in group 1 and 4 in group 2) who attended the post-treatment visit at the expected time but then lost to follow-up.

Of all those included, 11 were lost to follow-up (4 in group 1 and 7 in group 2) Demographic characteristics by groups are outlined in Table 1. The range of follow-up was >24 months in all patients.

**Table 1:** Demographic characteristics of the patients.

**Parameter**	**Group 0**	**Group 1**	**Group 2**	***P***
Sex ^§^				0.923
Female	4 (66.7%)	16 (57.1%)	12 (63.2%)	
Male	2 (33.3%)	12 (42.9%)	7 (36.8%)	
Age (years)^†^^^¥^^	58 ± 11	65 ± 13	64 ± 13	0.5401
Affected ear^§^				0.155
Left	5 (83.3%)	12 (42.9%)	7 (36.8%)	
Right	1 (16.7%)	16 (57.1%)	12 (63.2%)	
Number of crisis in the last 6 months^†^^^¥^^	8 ± 8	11 ± 5	13 ± 9	0.3345
Time since last crisis (days)^*^^^¥^^	5(2:7.5)	9(3:14)	5.5(3:9)	0.3618
Duration of disease (years)^*^^^¥^^	6(2:8)	9(6:16)	10(6:22)	0.1506
Migraine §	1 (16.7%)	4 (14.3%)	0	0.220
Tumarkin §	0	7 (25%)	7 (36%)	0.633
Stage §				**0.015**
Stage I	2	0	0	
Stage II	0	0	2	
Stage III	4	18	13	
Stage IV	0	10	14	
Mean follow up (months)^*^^‡^		51(30.5:62.5)	42(35:54)	0.2829

* *For not normally distributed data, median, 25th and 75th percentiles are shown*.

† *For normally distributed data, mean and standard deviation are shown*.

¥ *P < 0.05 is considered significant in the one way ANOVA test*.

‡ *P < 0.05 is considered significant in the Wilcoxon test*.

§ *P < 0.05 is considered significant in the Fisher's test*.

*P, significance value for the difference between groups*.

There were no significant differences between the three groups of patients regarding sex, age, affected ear, number of spells in the 6 months before treatment, time since last vertigo attack, disease duration, migraine diagnosis, presence of Tumarkin's crisis, or time of follow-up. Regarding the audiometric stage, there was a greater proportion of patients in more advanced stages in the treatment groups with respect to the untreated group (*p* = 0.015). The mean VOR gain after head impulses in the plane of each of the six semicircular canals showed no differences between the three groups of patients when analyzed at baseline in group 0 and before treatment in groups 1 and 2 (Table 2).

**Table 2:** Mean VOR gain and standard deviation according to ear and canal stimulation by groups before treatment.

		**Symptomatic ear**			**Asymptomatic ear**		
		**Superior**	**Horizontal**	**Posterior**	**Superior**	**Horizontal**	**Posterior**
Baseline	Group 0	0.75 ± 0.08	0.88 ± 0.07	0.72 ± 0.14	0.89 ± 0.06	0.97 ± 0.04	0.69 ± 0.11
Pre-ITG injection	Group 1	0.84 ± 0.17	0.98 ± 0.15	0.72 ± 0.12	0.88 ± 0.13	1.03 ± 0.13	0.78 ± 0.14
Pre-ITG injection	Group 2	0.81 ± 0.16	1.0 ± 0.19	0.78 ± 0.14	0.91 ± 0.17	1.03 ± 0.17	0.78 ± 0.14
		*P* = 0.509	*P* = 0.377	*P* = 0.471	*P* = 0.846	*P* = 0.739	*P* = 0.567

*Mean data and standard deviation are presented*.

**Figure 1:**
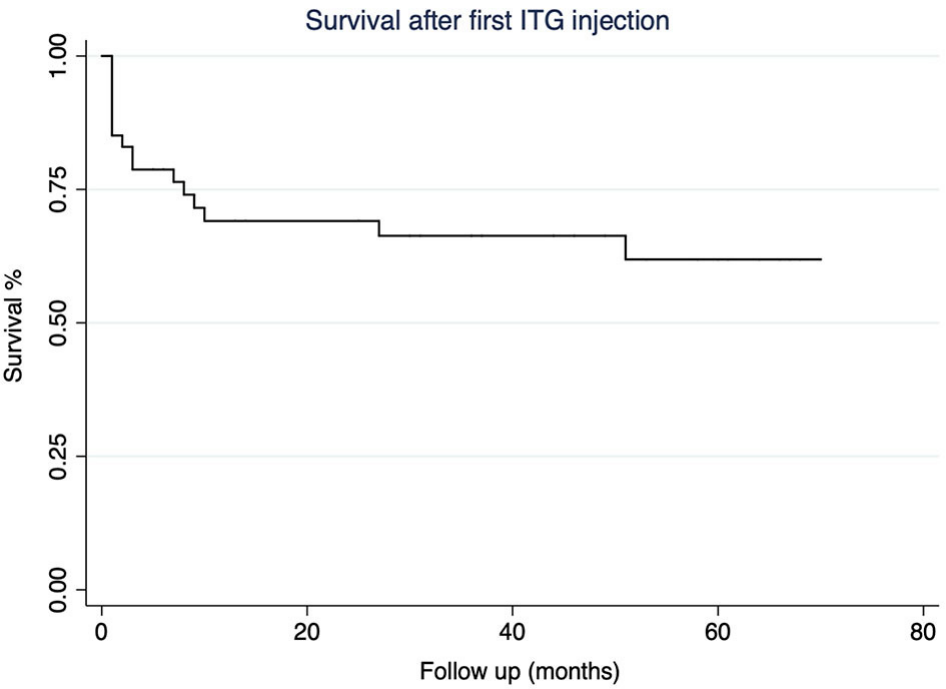
The Kaplan-Meier survival curve according to the necessity for a second intratympanic gentamicin (ITG) injection.

A survival curve represents a follow-up of treated (groups 1 and 2) patients (Figure 1). Twenty-eight patients received one single dose of ITG (59.6% of treated patients, group 1), whereas 19 received more than one ITG injection: 12 patients needed 2, 1 patient needed 3 ITG, 5 patients needed 4 ITG, and 1 patient needed 5 ITG. The mean time interval between the first and the second injection was 10 months (CI_95%_ 3–16, range 1–51), 5 months (CI_95%_ 0,7–10, range 1–18) between the second and the third injection, 10 months (CI_95%_ 0–24, range 1–46) between the third and the fourth injection, and 1 month between the fourth and the fifth ITG injection (1 subject). Among the patients receiving 4 injections, in 2 of them, an exploratory tympanotomy was further performed for direct application of gentamicin to the round window. In both patients, no fibrosis was found in the round window niche which was otherwise normal and access to the membrane was uneventful, and, in both patients, vertigo symptoms resolved after the procedure.

After ITG, 5 patients had to follow a vestibular rehabilitation treatment: 2 (7.1%) in group 1 and 3 (15.7%) in group 2. They mentioned chronic disequilibrium severe enough to limit their activities although there were no more vertigo crises. Those in group 1 had a very intense reduction in the gain of the VOR that affected the three semicircular canals: superior semicircular canal by 44 and 40%, horizontal canal by 82 and 78%, and posterior canal by 50 and 54%, respectively, for each patient. They were each treated 2 and 3 months, respectively, after the first follow-up visit. Those in group 2 were patients that each needed 2 injections (for 2 patients) and 3 injections (for 1 patient). The treatment took place 4 months after the last injection in all three cases.

### Results in Group 0

We found that after 1 month and in a stable clinical status (no new vertigo spell), the gain of the VOR showed very small modification as what occurred in the non-affected ear. It is, however, of interest to note an increase of the mean gain of the VOR close to 20% in the case of the anterior canal in the affected ear. The change in mean PTA both in the affected and unaffected ear was not significant and, in all of them, the difference from baseline to 1 month after the initial visit was <10dB. Particularly, it was worse in group 2 and better in group 4.

### Analysis of the Effect of ITG on the Gain of the VOR

**Figure 2:**
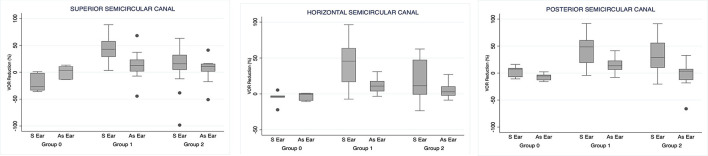
Vestibulo-ocular reflex (VOR) reduction 1 month after the first ITG injection. S Ear: symptomatic ear. As Ear: asymptomatic ear.

The change in the gain of the VOR in the first follow-up visit (1 month after ITG treatment), with respect to the pre-treatment value, is shown in Table 3 and Figure 2. The amount of reduction of the VOR gain, in the case of the superior and horizontal semicircular canals, was found to be significantly higher in group 1 than in group 2. Both groups had significantly higher gain reductions compared to the non-treated symptomatic ear (group 0). However, in the case of the posterior semicircular canal VOR gain, in groups 1 and 2, the reduction was equally high, but significantly different from the non-treated group (*p* = 0.133; Cohen's *D* = 0,41).

**Table 3:** Mean VOR reduction and standard deviation according to ear and canal after ITG injection.

	**Symptomatic ear**			**Asymptomatic ear**		
	**Superior**	**Horizontal**	**Posterior**	**Superior**	**Horizontal**	**Posterior**
Group 0	−19.29 ± 17.94	−5.27 ± 9.05	2.9 ± 11.47	0.17 ± 12.95	−3.17 ± 5.33	−6.96 ± 7.36
Group 1	43.14 ± 22.81	42.48 ± 28.11	42.35 ± 25.19	12.83 ± 21.57	11.13 ± 9.01	14.67 ± 14.01
Group 2	10.78 ± 37.55	20.0 ± 26.56	31.02 ± 30.93	5.33 ± 19.39	4.92 ± 9.85	−2.32 ± 22.44
	*P* 0 vs. 1 < 0.001	*P* 0 vs. 1 < 0.001	*P* 0 vs. 1 < 0.001	*P* = 0.489	*P* = 0.217	*P* 0 vs. 1 = 0.055
	*P* 0 vs. 2 = 0.019	*P* 0 vs. 2 = 0.006	*P* 0 vs. 2 = 0.014			*P* 0 vs. 2 = 0.664
	*P* 1 vs. 2 < 0.001	*P* 1 vs. 2 < 0.001	*P* 1 vs. 2 = 0.133			*P* 1 vs. 2 = 0.028

*Mean data and standard deviation are presented. VOR reduction data are presented in terms of percentage*.

**Figure 3:**
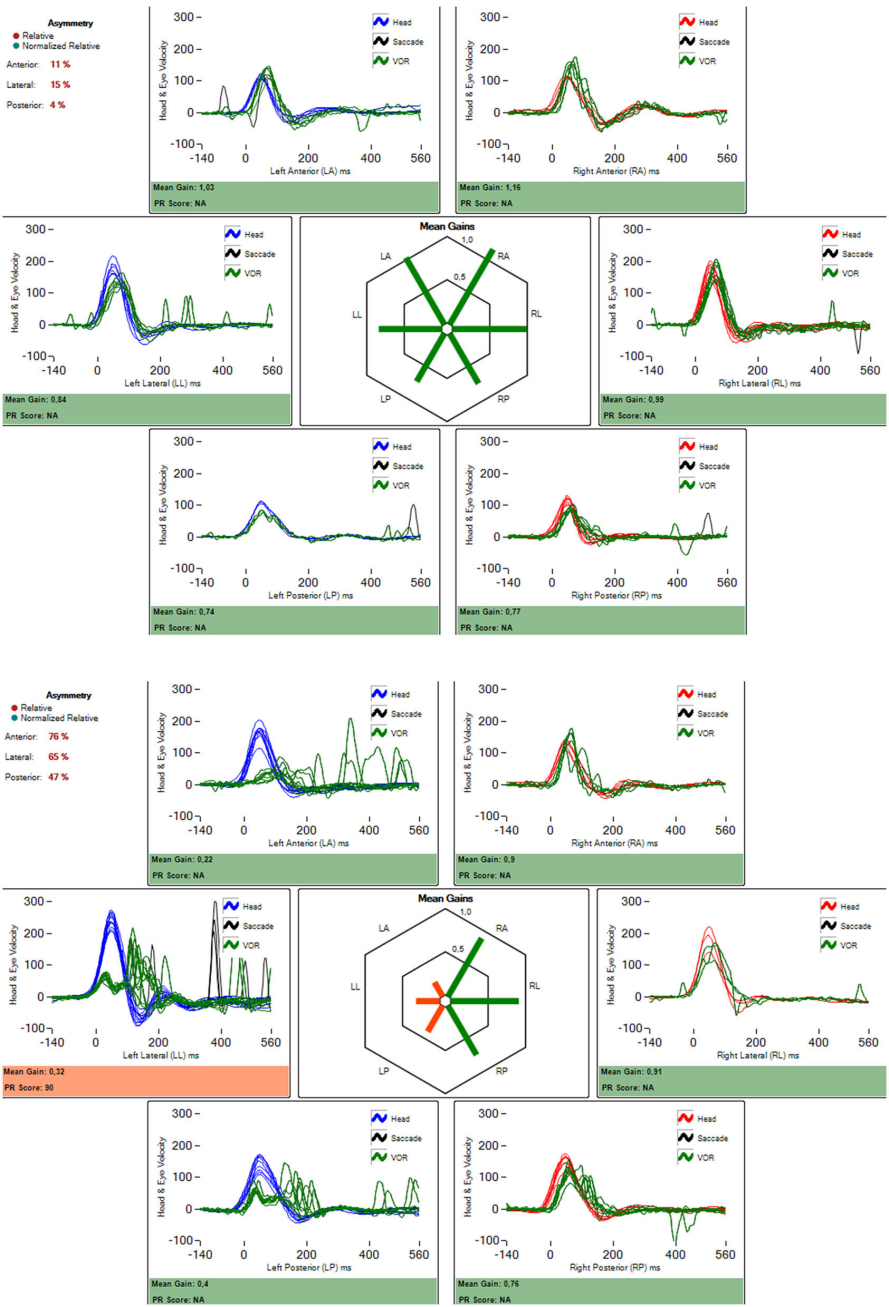
Video head-impulse test result in a patient with left ear Ménière's disease. In 3A before treatment with intratympanic gentamicin (1.1 ml of 27 mg/ml). In 3b, 1 month after the treatment. It is an almost complete damage to all three vestibular receptors in the corresponding ampullae. The amount of gain reduction (according to formula in the material and methods section) was 62%.

Figure 3 shows the traces of the video head impulse test of a patient belonging to group 1 before and 1 month after the first ITG injection in a subject with a significant reduction of the VOR after treatment.

In the asymptomatic (non-treated) ear, we found a small fluctuation in the value of the VOR gain in the case of head-impulses in the plane of the posterior semicircular canal such that the difference to pre-treatment values was significantly higher in patients in group 1 with respect to patients in the group 2. In addition, there was a trend toward significance with respect to group 0 but with a great size effect (Cohen's *D* = 1,64) (Table 3).

The ROC analysis for the reduction of the gain of the VOR of the horizontal semicircular canal of both treatment groups showed an AUC of.729 with a VOR reduction cutpoint of 11.6% (sensitivity = 89.29%; specificity = 52.63%; LR+ = 1.885; LR– = 0.204).

**Figure 4:**
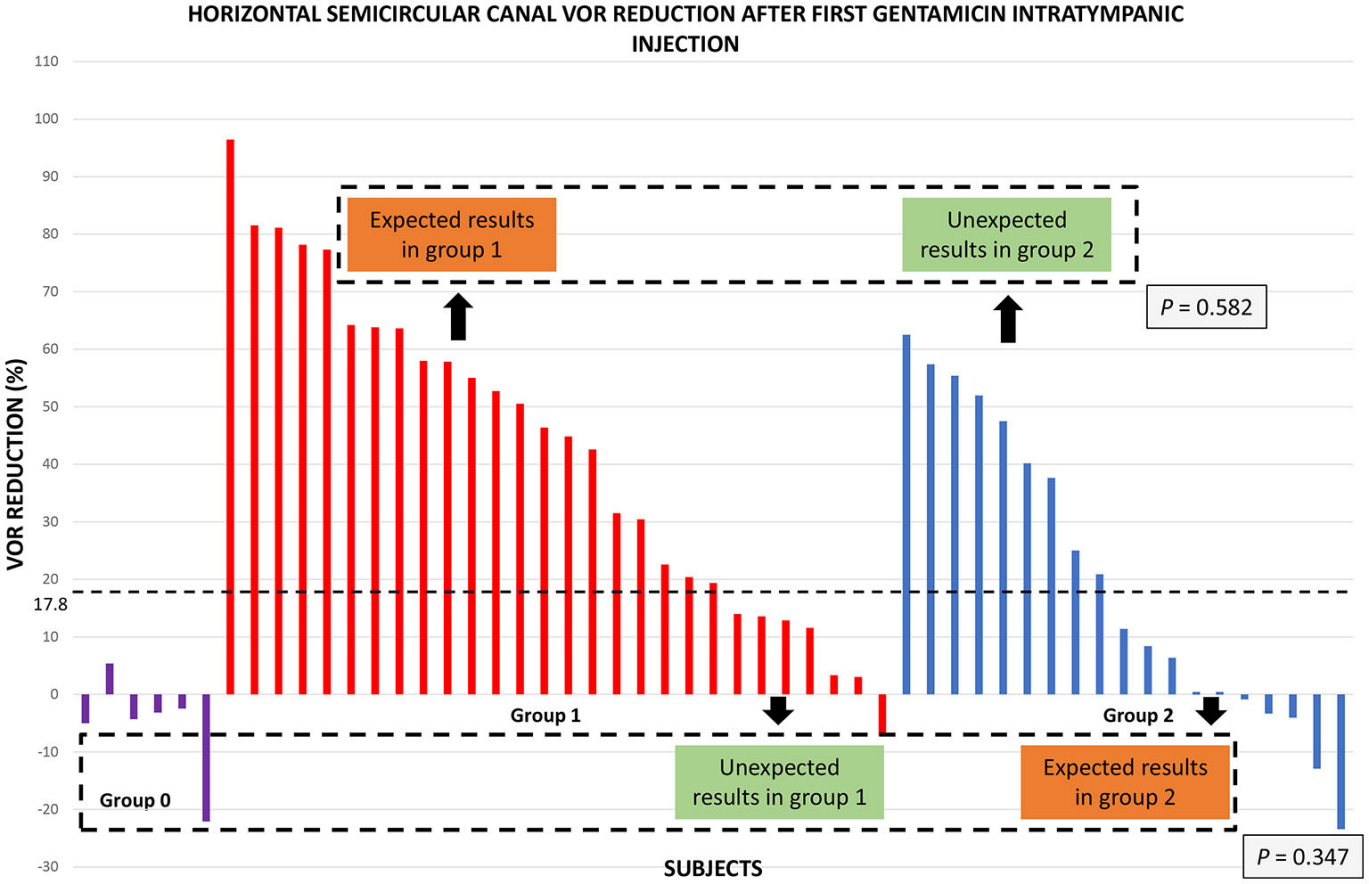
Horizontal semicircular canal VOR reduction 1 month after the first ITG injection. The bars represent the VOR reduction in every subject of the study. The dotted line represents VOR reduction cut point of 17.8% of the previous study which predicted the need for one or more ITG injections for vertigo control. According to this criterion, expected results in group 1 exceed this cut point, and unexpected results are below this cut point. On the other hand, unexpected results in group 2 exceed this VOR reduction value, and expected results are below this cut point. Note that VOR reduction values in the untreated group (group 0) are all of them below this cut-point. Dotted squares represent comparison between subgroups with VOR reduction above the cut-point and between subgroups below the cut-point and the control group (*P* values are located at the inferior right angle).

A more detailed analysis of the VOR gain reduction of the horizontal canal in each treatment group and follow-up is shown in Figure 4. In group 1, seven subjects (25%) were defined as “unexpected” and twenty-one subjects (75%) were defined as “expected.” In group 2, ten subjects (53%) were considered as “expected” and nine subjects (47%) were considered as “unexpected.” The VOR reduction of the “expected” and “unexpected” showed no statistically significant differences (*p* = 0.582), not even with group 0 (*p* = 0.347). Also, no differences were found regarding the number of expected and unexpected results in each treatment group (chi-square = 2.52; *p* = 0.112).

No differences were found between the subgroups of subjects with expected and unexpected results in both treatment groups or with the control group regarding age, sex, affected ear, number of crises before treatment, time since the last vertigo spell, duration of the disease, Tumarkin crises, migraine, or time of follow-up.

Further analysis showed that 2 subjects in group 2 showed no changes in the VOR gain, and 6 subjects (1 subject in group 1 and 5 subjects in group 2) showed an increase in the VOR gain in the horizontal canal of the symptomatic ear after the first ITG injection.

### Analysis of the Effect of ITG on Hearing

Regarding hearing as shown in Table 4, at the time of inclusion, PTA was slightly lower in patients in group 0 than in those in group 1 and showed a trend toward significance when compared with group 2 (*p* = 0.075; Cohen's *D* = 0,33). Interestingly, there were no significant differences between those in groups 1 and 2.

**Table 4:** Pure tone average before and after treatment and comparison of pure tone average between groups before and after treatment.

		**Symptomatic ear**	**Asymptomatic ear**			**Symptomatic ear**	**Asymptomatic ear**
Group 0	Baseline	40 ± 20	12 ± 9	Group 0	Baseline	40 ± 20	12 ± 9
	1 month control	37 ± 21	11 ± 9	Group 1	Pre-ITG injection	65 ± 20	28 ± 28
		*P* = 0.868	*P* = 0.868	Group 2	Pre-ITG injection	57 ± 17	19 ± 14
						***P*** **0 vs. 1** **=** **0.005**	*P* = 0.171
Group 1	Pre-ITG injection	65 ± 20	28 ± 28			*P* 0 vs. 2 = 0.075	
	Post-ITG injection	63 ± 17	29 ± 29			*P* 1 vs. 2 = 0.156	
		*P* = 0.868	*P* = 0.868				
				Group 0	1 month control	37 ± 21	11 ± 9
Group 2	Pre-ITG injection	57 ± 17	19 ± 14	Group 1	Post-ITG injection	63 ± 17	29 ± 29
	Post-ITG injection	58 ± 19	18 ± 11	Group 2	Post-ITG injection	58 ± 19	18 ± 11
		*P* = 0.868	*P* = 0.868			***P*** **0 vs. 1** **=** **0.003**	*P* = 0.072
						***P*** **0 vs. 2** **=** **0.029**	
						*P* 1 vs. 2 = 0.359	

*Mean data and standard deviation are presented (dB)*.

After the first ITG injection, the PTA of groups 1 and 2 was significantly higher than that of group 0 or not treated (Table 4). However, the PTA did not significantly change 1 month after treatment in any group (Table 4). The mean reduction in hearing in the treated ear was 1.3 dB in group 2, whereas in group 1 and in group 0, there was an increase of 0.14 and 2.3 dB, respectively. Eight subjects (4 subjects in each treatment group) showed a PTA modification >10 dB (17%), and 3 of them (2 in group 1 and 1 in group 2) progressed from a moderate to a severe hearing loss (6%). The changes in hearing in the asymptomatic ear were not significant.

## Discussion

The questions addressed here are all of interest when counseling patients with unilateral MD to whom we plan a treatment with gentamicin given intratympanically. A low-dose protocol has proved to be an effective method for controlling vertigo attacks with a low rate of hearing loss or persistent imbalance (2, 4, 16–18).

Our study first shows that 59.6% of the patients achieved complete vertigo control after one single dose of ITG after a prolonged follow-up period of up to 6 years, which is an important contribution of this report. This finding is in accordance with previously observed results (6–8).

The main problems when trying to predict an individual outcome in terms of the need for one or several injections in ITG treatments are the unpredictable effect of the drug and the unpredictable recurrence of vertigo after any nonsurgical and sub-ablative treatment. The rationale of this approach is to avoid side effects of unneeded treatments and maximize class A results (“the patient did not suffer any definitive vertigo spell in the 6 months prior to the date of follow-up evaluation,” according to the AAO-HNS) but also to provide sufficient information to clinicians and patients about the possible clinical course after a given number of injections. In this way, patients could better understand their own disease and expected prognosis after the treatment. All of which contributes to better functioning and well-being during follow-up (8).

There are a limited number of studies assessing the changes of the VOR measured by the quantitative head-impulse test in patients with MD after ITG treatment. Carey et al. observed a correlation between the reduction of the VOR gain as measured with the scleral search coil after fast head impulses and the reduction in the number of vertigo spells (3). Later, Lin et al. observed a significantly greater VOR reduction in the symptomatic horizontal canal after the first ITG injection in the single treatment group than in the multiple treatment groups (11). These findings are in accordance with the second result of our study: we found a significantly greater VOR gain reduction in all the canals in the treated groups than in the untreated group, and in the superior and horizontal canal of the affected ear in the single-dose group than in the group requiring more injections 1 month after the first ITG injection. Many authors have stated that a sub-ablative effect will maintain an optimal relationship between vertigo control and avoidance of side effects such as hypoacusis or chronic disequilibrium. Indeed, such vestibular function preservation may be important in case of subsequent development of bilateral MD.

Given the previously mentioned evidence, the corollary question is whether the amount of change in the gain of the VOR has any significance in clinical follow-up. Despite this, our results do not fully support that purpose, and we shall cover this topic in the next paragraphs.

According to our previous work, we can take into account as the endpoint of treatment at least a 17.8% of VOR gain reduction in the horizontal semicircular canal. In doing that, we found that 75% of patients in group 1 showed a gain reduction above this cut point. On the contrary, group 2 results are more heterogeneous with only 53% of subjects showing a reduction under this value. These discrepancies between the results and the observed clinical course in the present study explain the fair accuracy of the VOR gain reduction (AUC = 0.729) to correctly identify subjects who will have vertigo control with one-single dose of gentamicin and to predict the need for more treatment. Nguyen et al. observed an association between a horizontal canal VOR reduction >60% (clearly higher than our data) and a lower vertigo rate after the first round of treatment with ITG injections. However, they did not find any association between vertigo rate and VOR gain when those values were treated as continuous variables (19). Lin et al. (11) previously reported that partial vestibular ablation appeared to be beneficial by showing that patients who did not suffer vertigo recurrence had significantly greater decreases in ipsilateral horizontal VOR gain 1 year after treatment than those who had recurrent vertigo.

The differences between the findings in our previous study and our current results might be explained by the limited number of subjects in each group of treatment and by the shorter period of follow-up in the former report, since the probability of vertigo recurrence and the probability of needing an additional gentamicin injection increases in time. We have observed a patient who required a second injection after a symptom-free period of more than 4 years from the first treatment that is in accordance with Quaglieri et al. who reported that 10% of patients who showed an initial vertigo-free interval of at least 2 years presented a recurrence of vertigo spells after a symptom-free interval up to 5 years (6). However, in our subjects, the mean interval between the first and the second treatment was 10 months, i.e., before the recommended period of follow-up according to the AAO-HNS. We can thus speculate that our observations would not have been different with a shorter period of follow-up but still meet the AAO-HNS follow-up period criteria. In that study (6), they also found that the subgroup of *non-responder* subjects showed vertigo recurrence in the early stages of the treatment, and this can also be observed in our study according to the steepness of the survival curve to the left. The prolonged period of follow-up tries to overcome one of the main drawbacks of studies as it has been shown that vertigo recurrence rates may vary according to the duration of follow-up and that it can recur even after a symptom-free period lasting more than 2 years as said before (6–8). Another important difference relates to the number of patients included in the study. Other researchers found that a small modification (post-treatment to pre-treatment value) in the gain of the VOR for horizontal canal stimulation toward the affected and treated ear was significantly related to the need for further treatments (20). However, the number of patients included in that study was very low compared to ours. The differences in methodology, the age of the population, and disease duration may explain the differences in outcome. Further studies with a broader population and longer follow-up may address this issue.

The previously mentioned spontaneous rate of vertigo remission in MD might explain the cases with complete vertigo control where the VOR gain did not change after receiving a single ITG injection, namely, the patients included in what we have called “unexpected” and are in group 1. Another explanation for this finding could be the supposed damage to the dark cells by gentamicin which would lead to a decrease in endolymph production, limiting the hydrops, and subsequent vertigo (21). Pender described dark cell damage in the vestibular end-organs following gentamicin tympanolisis (22). However, a recent study using electron microscopy did not report significant damage to these cells in the semicircular canals of guinea pigs after a single dose of gentamicin (23).

In the case of those patients with an “expected” result but in group 2, the reasons why ITG does not produce any effect in canal function might be related to the delivery technique, drug concentration, patient positioning, round window obstruction by a second membrane as has been shown by Alzamil et al. (24), or an air bubble, round window membrane thickness, gentamicin loss through the eustachian tube, and inner ear membrane changes due to endolymphatic hydrops that limit diffusion of the drug as previous authors have stated (25).

Finally, as it has been shown by others and in this study, there is a subgroup of subjects who display a significant horizontal VOR reduction after a single dose of gentamicin and, however, continue having vertigo spells that needed more ITG. They are in group 2 and are called “unexpected.” This is not completely unexpected as a variable amount of canal function is preserved after intratympanic gentamicin therapy, unlike cases of surgical vestibular deafferentation. We did not assess the severity of vertigo episodes after the treatment, which is an important issue because patients often experience milder episodes following ITG injections. However, our outcome was the presence or absence of spontaneously appearing vertigo episodes after a single dose of gentamicin no matter the severity of the spells, not positional. In these patients, we must also take into account the possibility of spontaneous gain fluctuations related to the disease more than to treatment as can be shown in the affected ear of non-treated patients. That was found not to be homogeneous as we found to be very relevant for the anterior semicircular canal and much lower for the horizontal and posterior. As we relied only on the gain of the horizontal canal, this would preclude a merely causal relationship between the change in the gain and follow-up of the patient.

In our study, the mean VOR gain reduction 1month after the first ITG injection was greater in the single treatment group than in the multiple-treatment group. However, when we consider only the results of subjects with a VOR reduction >17.8% in both treatment groups, we did not observe any statistically significant differences between them. Likewise, when we compare the VOR gain change of the untreated group with the results of subjects having a reduction of the gain of the VOR below 17.8% in both treatment groups, no statistically significant differences were found between them. These observations and our previous results suggest that a VOR gain reduction >17.8% is associated with a good control of vertigo attacks in the first 2 years of follow-up, but not with a particular clinical outcome in the long-term, i.e., the need for one or more intratympanic gentamicin injections to achieve a complete vertigo control.

Besides the semicircular canal function, other variables might account for the reappearance or persistence of vertigo episodes. The question raises about the role of the otolith organs in vertigo episodes in MD, which was not evaluated in the present study. Absent VEMP has been reported in 35–54% of affected ears in MD (26), and a dissociation between oVEMP and cVEMP has been observed during MD attacks compared to quiescence (27). Following ITG therapy, both animal and human studies have shown damage to the otolith organs. In this sense, Liu et al. (28) obtained sensitivity and specificity of 93.5 and 66.7%, respectively, for significant control of vertigo (class A and class B) when combined a positive clinical head-impulse test and abolished cVEMP responses, suggesting the use of this combination as an end-point of treatment of low-dose ITG therapy. Others suggest the association between the persistence of VEMP responses after ITG injections and vertigo recurrence (29).

The possibility to *in vivo* observe endolymphatic hydrops is one of the major achievements in MD in recent years (30). Findings in the MRI are also being considered as an explanation for a reduced response to gentamicin as suggested by Marques et al. (31), such that different degrees of hydrops could indicate corresponding modification of perilymphatic dynamics in the vestibule.

We were also interested in the non-affected or asymptomatic ear. The VOR gain was also reduced in the asymptomatic ear in all the canals in group 1, and, to a lesser degree, in the superior and horizontal canal in group 2 which was also observed by others (3, 12). Büki et al. (32) ascribed this phenomenon to the diminished disinhibition coming from the treated ear through the commissural inhibitory fibers in a similar fashion as in cases of vestibular neuritis described by Weber et al. using scleral search coils (33). However, unlike cases of vestibular neuritis where the contralateral ear is supposed to be a “healthy” one, in MD there is increasing evidence supporting the fact that the asymptomatic ear is not always completely normal. There are histopathological and electrophysiological reports suggesting endolymphatic hydrops in the contralateral ear (34–36). Finally, Pyykkö et al. reported the presence of endolymphatic hydrops in 65% of contralateral ears of subjects with clinically unilateral MD as assessed with gadolinium-enhanced MRI (37), and we also in cases with fluctuating auditory or vestibular symptoms (38). Contrary to these hypotheses (the push-pull mechanism between co-planar canals through the commissural system or subclinical disease) is our finding that the modification in the gain of the VOR in those ears is not significantly different to the spontaneous modifications as shown when comparing to results in group 0. The number of included patients in that group is small, but findings agree to those recently reported in normal subjects and in patients with MD in whom the gain of the VOR varied little over very short (2 days) periods of time (39), which supports the high test-retest reliability of the measurement (40). This is contrary to what has usually been shown close to a new vertigo crisis (41).

The low overall hearing reduction observed in the treatment groups is our fourth result. It is similar to that observed by Cohen-Kerem (18), probably due to the low cochlear gentamicin levels produced by one-shot application protocols as has been estimated by Salt et al. using a computer simulation program (17). This is an argument for the safety of the use of ITG when indicated and correlates with similar protocol findings (42).

Our work also supports the use of gentamicin according to an on-demand protocol that limits further unneeded treatments based on the good control of vertigo and limited hearing damage but also of the reduced amount of unsteadiness because of the procedure. Although numbers are very small, they replicate the findings of chronic disequilibrium in patients with complete control of vertigo obtained when several treatments (group 2) were performed (5). Interestingly, when that procedure was followed (weekly injections until modification of the results of the vestibular bedside test) no difference was found between patients that needed subsequent vestibular rehabilitation regarding age, pre-treatment PTA and canal paresis, and number of gentamicin injections (43). Now, we have shown that when control of vertigo attacks is obtained with 1 single injection, the number of those that need vestibular rehabilitation is less. Also, that the change in the value of the gain of the VOR from pre-treatment to post-treatment value was very intense in these 2 patients. To further characterize this follow-up, a more in-depth study needs to be performed considering at least other variables as the amount of change induced by gentamicin in the otolithic maculae as measured by VEMP.

## Limitations

This study has some limitations. First, as a retrospective study, there is a risk of selection bias. The prolonged follow-up period in many patients increases the risk of recall bias for the outcome of vertigo recurrence as it is sometimes difficult to remember whether there was a true vertigo episode. Thus, a misclassification of subjects into the wrong group could occur. Nevertheless, if this situation was true, more patients in the subgroup of expected results in group 1 would belong to a subgroup of unexpected results of group 2, supporting the fact that the VOR gain reduction cannot predict the clinical outcome. Second, we have used a measure for cutoff analysis obtained in a previous study. In the actual one, some of those patients are included, and this represents a bias in the analysis that should be taken into account. Third, the lack of information on the vertical canals in 10 subjects has precluded a detailed analysis of the horizontal canal. Very recently, it has been shown that there is a differential effect according to the semicircular canal evaluated such that the reduction in the gain of the VOR was more intense for the posterior and horizontal canal than for the superior semicircular canal (18). Fourth, we did not assess VEMP responses during the vestibular work-up which may bring information about the reasons for the vertigo recurrences in subjects with significant VOR reduction after ITG injections. Finally, another weakness in the present report is the small number of untreated subjects of group 0 because of the difficulty to recruit untreated definite subjects with MD, although the size effect was significant only when comparing the posterior canal VOR reduction between group 0 and group 1 in the asymptomatic ear (Cohen's *D* = 1,64). For this reason, these results should be considered with caution.

## Conclusions

Our results show that one single dose of intratympanic gentamicin allows for a good vertigo control in the long term with similar effectiveness to that reported by others. Compared to the untreated group, the mean VOR gain reduction of the horizontal and superior canals, as measured with the vHIT 1 month after the first injection, was significantly higher in the treatment groups. A similar finding was obtained when comparing the single-treatment group and the multiple-treatment group, where the reduction in the former was significantly higher. However, the detailed analysis of the horizontal canal VOR reduction shows that, during the follow-up, many patients do not display the expected clinical course according to our previous endpoint of treatment. This explains the only fair accuracy obtained for the horizontal canal VOR reduction, as measured 1 month after the first injection, in predicting the need for no further instillations in the long term. In other words, a significant reduction of the VOR gain after the first ITG injection is less accurate in predicting the need for only one single dose for complete vertigo control than previously reported. A prospective study with a larger number of subjects and serial VOR assessment following ITG therapy might contribute to better characterizing the effect of gentamicin on canal function in time and help understanding the discrepancy between VOR reduction and vertigo control in the long term.

According to our observations, the protocol of low dosage intratympanic gentamicin as needed has proved to be a safe way to control vertigo attacks with a low risk of hearing loss.

## Data Availability Statement

The original contributions presented in the study are included in the article/supplementary material, further inquiries can be directed to the corresponding author.

## Ethics Statement

The studies involving human participants were reviewed and approved by Comite de Etica de la Investigación (CEI). The patients/participants provided their written informed consent to participate in this study.

## Author Contributions

NP-F, RW-V, and EM-S contributed to conception and design of the study. RW-V and DC-G organized the database. DC-G performed the statistical analysis. RW-V wrote the first draft of the manuscript. RW-V, NP-F, RM-H, EM-S, and PM wrote sections of the manuscript. All authors contributed to manuscript revision, read, and approved the submitted version.

## Conflict of Interest

The authors declare that the research was conducted in the absence of any commercial or financial relationships that could be construed as a potential conflict of interest.

## Publisher's Note

All claims expressed in this article are solely those of the authors and do not necessarily represent those of their affiliated organizations, or those of the publisher, the editors and the reviewers. Any product that may be evaluated in this article, or claim that may be made by its manufacturer, is not guaranteed or endorsed by the publisher.
